# Circulating tumor cells in hepatocellular carcinoma: single-cell based analysis, preclinical models, and clinical applications

**DOI:** 10.7150/thno.48918

**Published:** 2020-10-26

**Authors:** Qian Zhang, Yuan Rong, Kezhen Yi, Lanxiang Huang, Ming Chen, Fubing Wang

**Affiliations:** Department of Laboratory Medicine, Zhongnan Hospital of Wuhan University, Wuhan 430071, P. R. China.

**Keywords:** circulating tumor cells, single-cell based analysis, preclinical models, hepatocellular carcinoma, clinical application

## Abstract

Circulating tumor cells (CTCs) are shed into the bloodstream from primary tumors and metastatic lesions and provide significant information about tumor progression and metastasis. CTCs contribute to tumor metastasis through the epithelial-to-mesenchymal transition (EMT). CTC clusters and stem-like phenotypes lead to a more aggressive and metastatic potential. CTCs retain the heterogeneity and imitate the nature of corresponding primary tumors. Therefore, it is important to use single-cell based analysis to obtain information on tumor heterogeneity and biology. CTCs are also good candidates for building preclinical models (especially 3D organoid cultures) for drug screening, disease modeling, genome editing, tumor immunity research, and organ-like biobank establishment. In this article, we summarize the current CTC capture technology, dissect the phenotypes associated with CTC metastasis, and review the progress in single-cell based analysis and preclinical modeling of the pattern and kinetics of CTCs. In particular, we discuss the use of CTCs to assess the progression of hepatocellular carcinoma (HCC).

## Introduction

Circulating tumor cells (CTCs), which carry valuable tumor information, arise from the hematogenous diffusion of metastatic tumors 1 and have always been the focus of clinical research. In the past decades, various CTC capture technologies have been developed based on the biological features of CTCs, such as immune affinity and physical characteristics. In breast, prostate, and colorectal cancers, using the FDA-approved CellSearch platform, the CTC numbers have been correlated with progression-free survival (PFS) and overall survival (OS) 2, 3.

With emerging technological developments, CTC research is not limited to enumeration. Different CTC phenotypes have been analyzed, including the epithelial, mesenchymal, and stem cell types and CTC clusters, which were associated with distinct kinetics and functions 4. In particular, advances in sequencing technology have facilitated the analysis of the genomes, transcriptomes, and proteins of individual CTCs, deepening our understanding of tumor heterogeneity for companion diagnostics 5, 6. Besides, *in vitro* and *in vivo* CTC culture approaches have provided significant insights into tumor development and metastasis 7, 8.

Hepatocellular carcinoma (HCC) is the seventh most common cancer and the second leading cause of cancer-related deaths worldwide 9. Current therapies for HCC include radical surgical resection, local ablation, or liver transplantation, which only apply to early-stage HCC, and their efficacy is not satisfactory due to the high recurrence rate 10. Therefore, the approaches for diagnosing and monitoring HCC are important. Currently, diagnosing and monitoring HCC mainly depend on serum biomarker detection, biopsy, and imaging analysis. Each of these approaches suffers from drawbacks. Almost one-third of HCC patients show no significant changes in alpha-fetoprotein (AFP), an important serum biomarker 11. A biopsy is invasive and may cause injury to patients and does not apply to dynamic monitoring. The sensitivity and specificity of medical imaging are low when a lesion is less than 2 cm.

In recent years, a series of “liquid biopsy” techniques have been developed to evaluate HCC biomarkers 12. Liquid biopsy uses a non-invasive approach to obtain tumor information for tumor diagnosis and dynamic monitoring by assessing CTCs, circulating tumor DNA (ctDNA), microRNA (miRNA), and extracellular vesicles (EVs). CTCs obtained from HCC patients represent live tumor cells, which carry more comprehensive tumor information. Therefore, it is necessary to retrospectively analyze the clinical application of CTCs in the diagnosis and treatment of HCC.

## Strategies for CTCs enrichment

**Figure 1:**
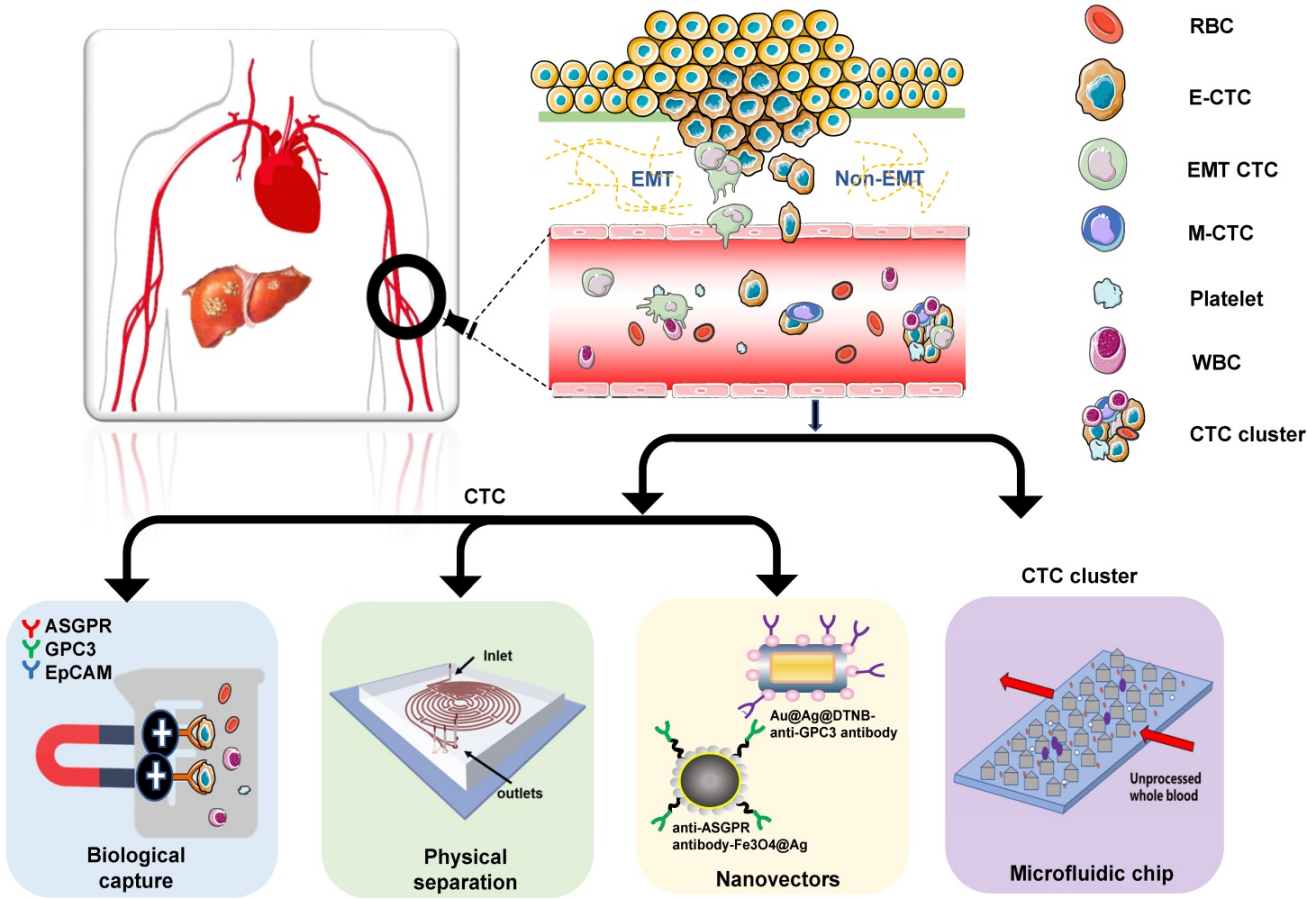
Overview of CTC capture techniques: CTCs are obtained from patients' blood samples in a non-invasive way. Many techniques distinguish CTCs from normal blood components (mainly red blood cells and white blood cells). HCC CTCs are primarily isolated based on their unique biological or physical properties. Currently, nanomaterials and microfluidic chips are widely used for CTCs capture.

CTCs, first discovered by Thomas Ashworth 150 years ago, can be obtained multiple times from tumor patients without an invasive approach. The recent development of new and powerful technologies has remarkably facilitated the precise capture of CTCs 13. Current CTC capture techniques are summarized in** Figure 1**.

CTCs from HCC patients are primarily isolated based on their unique biological or physical properties. The biological methods include immune magnetic bead capture and nucleic acid aptamer capture. Court *et al.* 14 optimized HCC CTC capture using the NanoVelcro method that recognizes several cell-surface markers, such as asialoglycoprotein receptor (ASGPR), glypican-3 (GPC3), and epithelial cell adhesion molecule (EpCAM). In this study, HCC-CTCs were identified in 97% of HCC, and accurately discriminated HCC and non-HCC patients. Wang et al. used sLex-AP (aptamer for carbohydrate sialyl Lewis X) to coat the HA/CTS nanofilm to identify HCC CTCs in 59.5% of HCC patients 15. These studies confirmed that both the CTC positive rate and number significantly correlated with tumor size, portal vein tumor thrombus, and the tumor-node-metastasis (TNM) stage. The physical properties applicable to CTC detection include size 16, 17, density 18, deformability, migratory capacity 19, and electric charge 20. Wan *et al.* developed a novel Labyrinth microfluidic device, offering unique features over other inertial devices to efficiently isolate CTCs from the peripheral blood of HCC patients 21. It incorporates 56 sharp corners to allow focusing on smaller cells that were previously difficult to target. Using this device, CTCs were detected in 75% of patients with early-stage HCC (TNM 0/I) and 96.2% of patients with advanced-stage HCC (TNM II-IV).

Currently, nanomaterials and microfluidic chips are widely used for CTC capture. According to the study by Ozkumur *et al.*, “CTC-iChip”, an inertial focusing-enhanced microfluidic CTC capture platform, is capable of isolating CTCs with or without tumour membrane epitopes 22. This technique applies to virtually all tumors. Zhang *et al.* proposed an on‐chip strategy combining multiplex SERS nano-vectors and multivariate analysis for *in situ* profiling of circulating tumor cell phenotypes on microfluidic chips 23. Besides, Pang *et al.* reported a magnetically assisted surface‐enhanced Raman scattering (SERS) biosensor for HCC CTC detection 24. This biosensor consists of anti‐ASGPR antibody‐Fe3O4@Ag magnetic nanoparticles and anti‐GPC3 antibody‐Au@Ag@DTNB nanorods. Because of the dual‐selectivity of the two antibodies, and the dual‐enhancement SERS signal of the MNP silver shell and the Au@Ag NRs SERS tags, a limit of detection of 1 cell/mL for HCC CTC in human peripheral blood with a linear relationship from 1 to 100 cells/mL can be achieved. The increasingly extensive research also proved the importance of CTC cluster analysis, and developed efficient CTC cluster technology. For example, a unique microfluidic chip, termed Cluster-Chip, which exploits the unique geometries of cellular aggregates to distinguish CTC clusters from single cells in the blood, was introduced by Sarioglu *et al.* 25. This technique enables specific and label-free isolation of CTC clusters from patients with different cancers. Although there are many emerging capture technologies. Non-label capture and in-situ inspection methods are still urgently needed to facilitate subsequent culture and single-cell analysis of CTCs.

## Metastasis-associated CTC phenotypes

### Epithelial-mesenchymal transition (EMT)

EMT is a process that initiates metastasis 26-28. Liu *et al.* showed that tumor cells undergo gradual or complete EMT (E, E/m, M/e, and M) that is associated with slow proliferation, loss of epithelial cell adhesion molecule EpCAM expression, and increased migration 29. In the metastatic cascade, tumor cells undergoing EMT have an increased ability to intravasate into the lympho-vascular system. Once tumor cells migrate into blood vessels, they become CTCs. E-type and E/m-type CTCs have enhanced capacity to adhere and extravasate to distant sites. Importantly, E-type and E/m-type cells are associated with higher proliferation and metastatic outgrowth, while M/e- and M-type cells are related to long-term tumor recurrence. In HCC, Liu *et al.* showed that mixed CTCs might be vital for intrahepatic metastasis, and mesenchymal CTCs could predict extrahepatic metastasis 30. Qi *et al.* revealed that total CTC count and the proportion of M-CTCs are negative factors for postoperative HCC recurrence 31. However, the spatial heterogeneity of phenotypic and molecular characteristics of CTCs within the circulatory system remains unclear. Therefore, Sun *et al.* mapped the distribution and characterized the biological features of HCC CTCs along the transportation route 32. Single-cell characterization demonstrated that the EMT status of CTCs was heterogeneous across different vascular compartments. CTCs were predominantly epithelial at release but switched to EMT-activated phenotype during hematogenous transit via the Smad2 and β-catenin pathways. Besides, EMT activation in the primary tumor correlated with total CTC number, rather than epithelial or EMT-activated subsets in hepatic veins (HV) 32.

### CTC clusters

Besides EMT, CTC clusters have been proposed to indicate the initiation of tumor metastasis 33. Although CTC clusters are rare in circulation relative to single CTCs, they have 23- to 50-fold increased metastatic potential 34, 35. It was first demonstrated by Aceto *et al.* that CTC clusters originate from oligoclonal tumor cell groupings rather than cell aggregation in the blood vessels 34. They also provided evidence that plakoglobin-dependent intercellular adhesion is crucial for the formation of CTC clusters. Sun *et al*. prospectively measured CTCs at five key vascular sites in patients with localized HCC 32. Circulating tumor microemboli (CTM) were detected in tumor efferent vessels in approximately half of the patients, indicating that CTM originate from oligoclonal tumor cell groupings. However, it is worth noting that CTM were identified in hepatic veins (HV) but not peripheral artery (PA) in 15 patients, whereas 5 of them exhibited reemergence of CTM in peripheral vein (PV). This suggests that singular CTCs might be able to reaggregate in the bloodstream, which is contrary to the conclusion drawn by Aceto* et al.* 36. They compared methylated DNA regions and found that CTC clusters and CTCs have distinctive DNA methylation status at differentially methylated regions (DMRs). The DMRs of CTC clusters feature hypomethylation at the binding sites of stemness- and proliferation-associated transcription factors, such as Oct4, Nanog, Sox2, and Sin3A 36.

### Stem-like phenotypes

It has been more than a century since Cohnheim proposed the “embryonic theory” of cancer. The cancer stem cell (CSC) hypothesis argues that cancers arise from a subset of malignant cells that possess stem cell characteristics 37. Although both CSCs and mature cancer cells can enter the bloodstream, CSCs are more prone to survive in the circulation and reside in distant organs or recirculate back to the liver 37. Currently, CD133 and CD90 have been used to distinguish and isolate CTCs or CSCs in HCC. However, so far, no good circulating tumor stem cell (CTSC) marker has been identified in HCC. Liu *et al.* showed that ICAM-1 is a marker of HCC stem cells. They quantified circulating CD45-ICAM-1+ tumor cells from 60 HCC patients using flow cytometry and found that higher frequencies of circulating CD45-ICAM-1+ cells in HCC patients correlated with more aggressive tumor behavior and worse clinical outcomes 38. Sun *et al.* found that EpCAM^+^ CTCs show stem cell characteristics and indicate poor prognosis of HCC after curative resection 39.

In animal models, CD90+CXCR4+ HCC cells could be CTSCs, as reported by Zhu *et al.* 40. Selective elimination of these cells may substantially improve the efficacy of current HCC therapy by reducing metastasis. The oncogene Sox12 may be a novel marker for CSCs in HCC as proposed by Zou *et al.* 41. Compared to Sox12- HCC cells, Sox12+ HCC cells were more frequently detected in circulation, had a higher efficiency of generating tumor spheres in culture or forming distal metastasis, and displayed greater chemo-resistance to cisplatin 41. Although there is no current consensus on the CSC phenotype in HCC, these studies provide a guideline for building a molecular panel to assess CTC stemness in clinical applications. Such a CTC panel, including four putative stem cell biomarkers (EpCAM, CD133, CD90, and CK19) was constructed by Guo *et al.* 42. The panel performed well in detecting early-stage and AFP-negative HCC, as well as differentiating HCC in hepatitis B infection (CHB), liver cirrhosis (LC), and benign hepatic lesions (BHL) 42.

Although the tumor metastasis mechanism remains unclear 34, CTC phenotypes can be classified to evaluate their malignant potential before and during tumor therapies. Wang *et al.* systematically investigated the clinical significance of diverse subtypes of CTCs and showed that the presence of EpCAM+ multiploid CTSCs (cut-off: ≥1 cell in 6 ml of blood), EpCAM- small triploid CTCs (≥5 cells), CTM (≥1), and increased triploid CTCs, were highly relevant for poor prognosis 4.

## Single-cell based analysis

**Figure 2:**
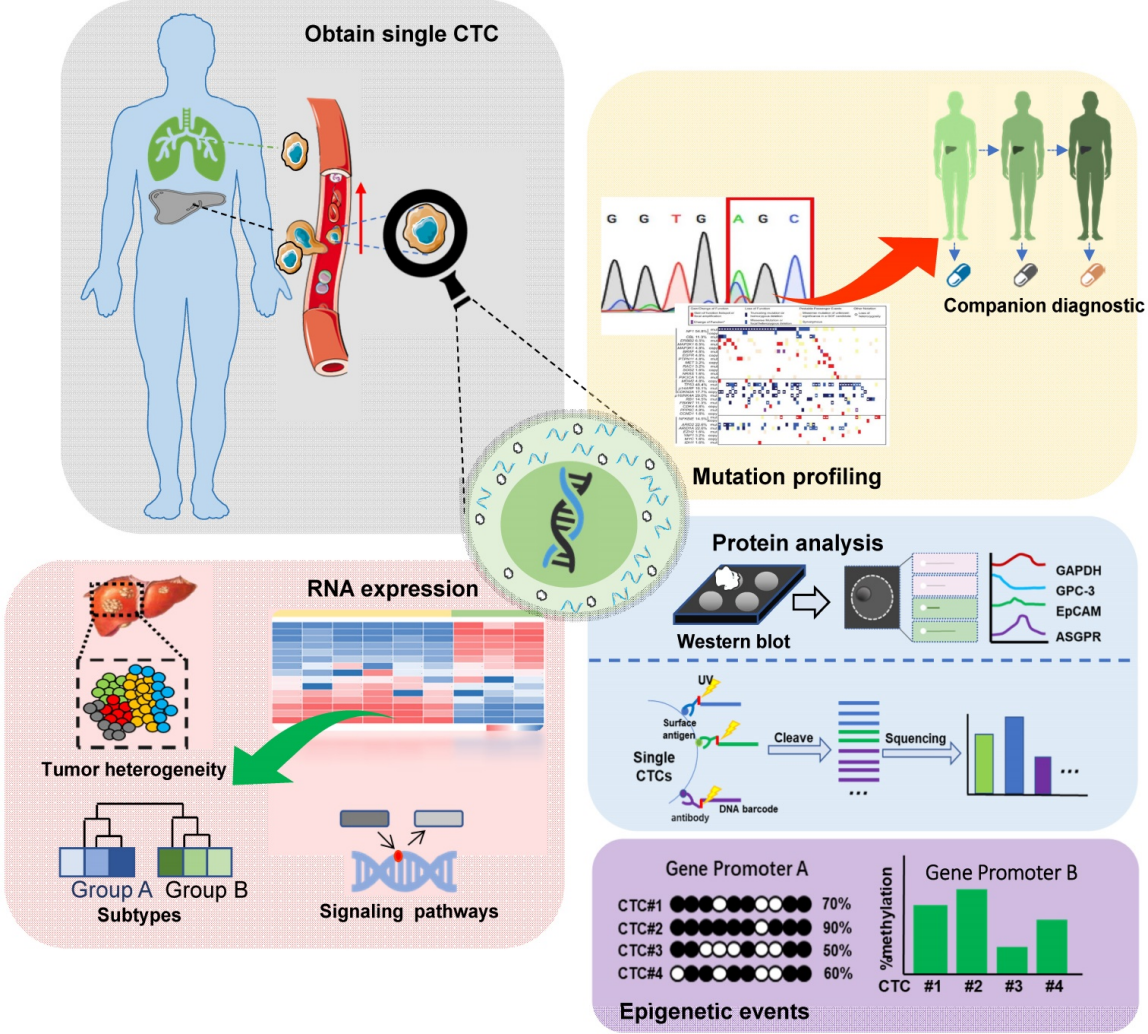
Schematic representation of single-cell analysis of CTCs in HCC: Genomic, transcriptomic, proteomic, and epigenetic analyses are included. In the genomic analysis, tumor-related gene mutations are analyzed for companion diagnostics. In the transcriptome analysis, RNA levels are analyzed to assess tumor heterogeneity, identify tumor cell subsets, and dissect signaling pathways related to chemo-resistance. The epigenetic and proteomic analyses offer additional information about tumor heterogeneity.

Single-cell sequencing technology has been a major breakthrough in sequencing history. CTCs can be processed and analyzed as single cells and then subjected to single-cell sequencing. Given the considerable heterogeneity of CTCs, it is essential to analyze the molecular and genetic characteristics of single CTCs 5, 6. The schematic of single-cell analysis of CTCs is shown in **Figure 2**.

### Single-cell genomic and transcriptomic analysis

With advances in the next-generation sequencing (NGS) and single-cell sequencing (SCS) technology, it is possible to obtain the complete genomes of CTCs and compare them with the corresponding primary and metastatic tumors. Several clinically relevant genomic alterations have been discovered, such as single nucleotide variations (SNV), microsatellite instability, and copy-number variations, providing valuable information for the companion diagnostics 43. At the transcriptomic level, gene expression profiles of individual CTCs can reveal complex expression patterns within and across patients. Importantly, chemo-resistant tumor cell subsets and relevant signaling pathways could also be identified 44, 45. D'Avola *et al.* developed a new technology combining image flow cytometry and high-density single-cell mRNA sequencing to identify CTCs in HCC patients 46. Genome-wide expression profiling of CTCs using this approach demonstrated CTC heterogeneity and detected known oncogenic drivers in HCC such as Oct4. In another study, Sun *et al.* collected blood from the peripheral vein, peripheral artery, hepatic vein, inferior hepatic vena cava (IHIVC) and portal vein (PoV) before tumor resection. They analyzed the EMT phenotypes of CTCs using the 4-channel immunofluorescence CellSearch assay and microflow quantitative RT-PCR. The study demonstrated the heterogeneity of EMT status in CTCs across different vascular compartments 32. CTCs were predominantly epithelial at release but switched to EMT-activated phenotype during hematogenous transit via Smad2 and β-catenin signaling. Thus, single-cell analysis provides a novel tool for biomarker identification in HCC and would ultimately help customize therapeutic interventions.

### Epigenetic analysis

Epigenetic events, including histone modification and DNA base modifications (such as methylation and hydroxy-methylation), also contribute to tumor cell heterogeneity. ChIP-seq (Illumina) is used for studying histone modifications at the single-cell level 47, and the whole-genome bisulfite sequencing (WGBS) offers the most comprehensive profile of DNA methylation 48. Pixberg *et al.* established a CTC line using the peripheral blood from a mouse HCC model 49. To explore the mechanisms regulating the expression of HGF and c-MET, DNA methylation at the promoters of both genes was evaluated using the bisulfite-conversion and high-resolution melting analysis. The overexpression of HGF and c-MET in CTCs was associated with decreased DNA methylation at their promoters. The study concluded that during hematogenous dissemination, HCC CTCs undergo EMT under the influence of up-regulated HGF. This process also involves the up-regulation of c-MET induced by demethylation at 6 CpG sites. So far, the epigenetic characteristics of CTCs remain largely unexplored. However, existing data strongly encourage epigenetic analysis of CTCs to understand the molecular mechanisms of HCC metastasis.

### Protein analysis

Single-cell protein analysis identifies the heterogeneity of seemingly similar tumor cells, providing critical insights into the mechanisms underlying tumor heterogeneity 50, 51. For this, Western blotting is the most popular protein analysis approach. Recently, multiple single-cell protein quantification methods have been developed. Flow cytometry has a high flux 52-54 but limited multiplexing capability due to the spectral overlap of fluorescent-labeled antibodies. By using the transition metal mass marker, mass spectrometry significantly improves (> 30) the detection of multiple proteins 55. However, these methods are ineffective in finding rare target cells, especially in the presence of overwhelming background cells 56, 57. Based on DNA barcoding and high-throughput sequencing, Wang *et al.* developed a microchip-assisted single-cell proteomic method for profiling CTC surface antigens facilitating the phenotypic and functional analysis of single CTCs 58.

## Preclinical models

Although the techniques mentioned above have provided abundant information about CTCs, their functions still need to be validated in preclinical models. CTC-based preclinical models include 2D cultures, CTC spheroids, CTC-derived organoids, and xenografts 8. Many studies have successfully established 2D cultures and CTC spheroids from cancer patients 59-62. For example, Zhang *et al.* isolated CTCs from 31 patients and cultured them into spheroids 63. Furthermore, the sensitivity of CTCs to chemotherapeutic agents (sorafenib or oxaliplatin) can be effectively tested utilizing the spheroids. 2D cultures and spheroid formation are easily conducted at low costs. However, cells generated by these two methods lack genomic and spatial heterogeneity. To address this issue, CTC-derived xenografts and organoids have emerged recently.

### CTC- derived xenograft (CDX)

CTC-derived xenograft (CDX) is an *in vivo* model in which CTCs enriched from patient blood are injected into immunocompromised mice to generate tumors and expand the number of original tumor cells 64. CDX is a good method for imitating tumor evolution and studying the metastatic process 65, 66. Baccelli *et al.* had developed a xenograft assay and shown that primary human luminal breast cancer CTCs contain metastasis-initiating cells (MICs) that cause metastasis in the bone, lung, and liver. These MIC-containing CTCs expressed EpCAM, CD44, CD47, and MET 67. In a small cohort of patients with metastasis, the number of EpCAM^+^CD44^+^CD47^+^MET^+^ CTCs correlated with increased metastatic sites and a poor survival rate. Recently, Vishnoi *et al.* established a novel triple- negative breast cancer (TNBC) liver metastasis‐specific CDX model that selectively recapitulates CTC biology for four sequential generations of mice 68. Using multi-parametric flow cytometry analysis, immune phenotyping, and genomic sequencing, 597 genes specific to TNBC liver metastasis were identified in isolated CDX‐derived cells. This study provided a remarkable insight into the mechanism of TNBC disease progression in the liver. CDX can not only study the metastasis process, but also mirror the response of small cell lung cancer patients to platinum and etoposide chemotherapy, providing tractable systems for therapy testing and understanding drug resistance mechanisms 69. Although CDX represents classical preclinical mouse models that are relatively easy to handle, CDX development still presents an enormous challenge due to low CTC prevalence in several cancers. The generation efficiency of CDX is much lower than that of patient-derived xenograft (PDX) 70. Currently, the CDX model has not been successfully established in HCC so continuous endeavor is still required.

### CTC-derived organoid (CDO)

**Figure 3:**
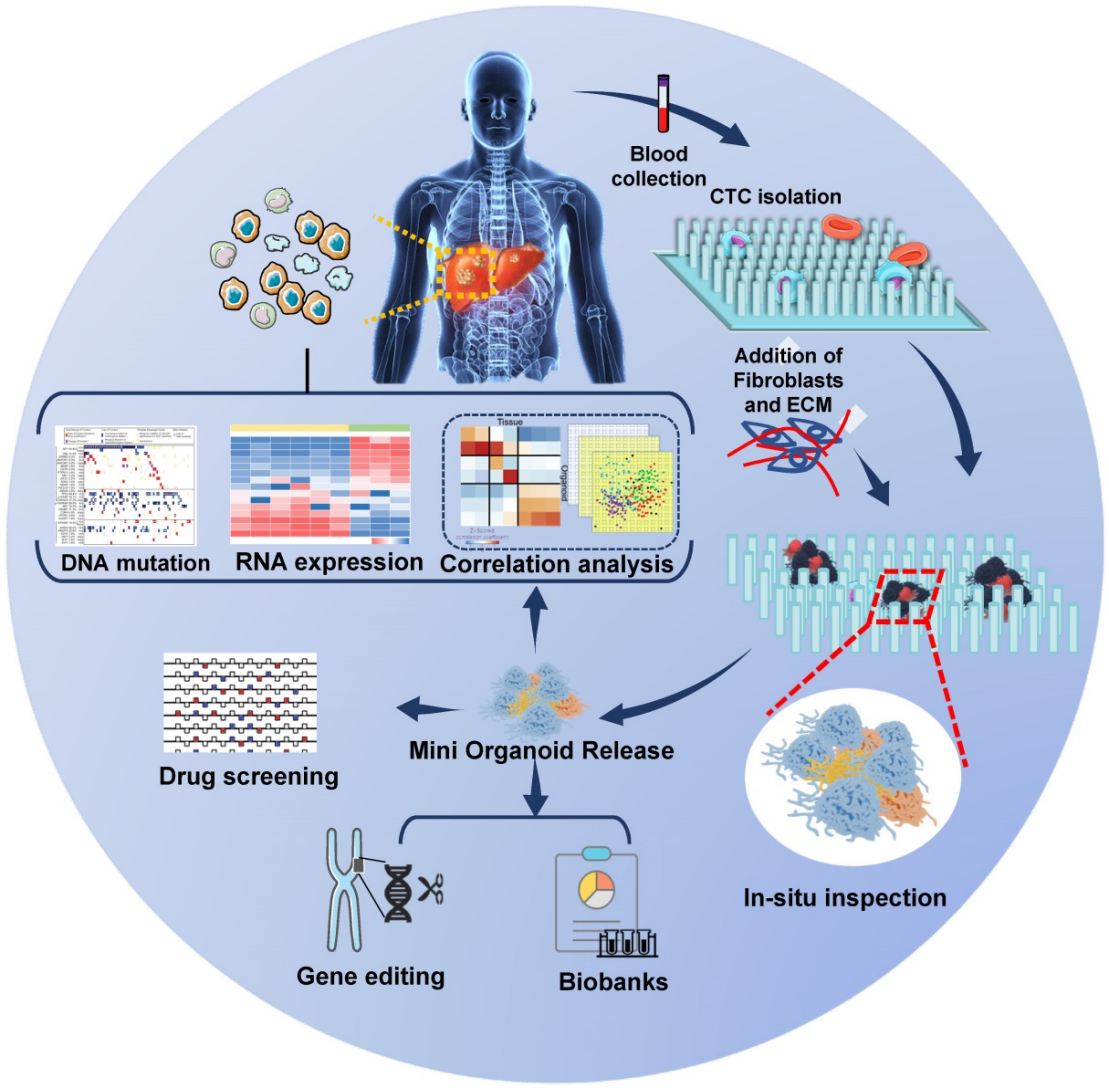
Speculative strategy for analyzing CTC-derived organoids in HCC: Patients' blood samples are collected, and CTCs are captured and cultured *in situ* on a specific chip to form mini organoids. The mini CTC-derived organoids provide *in situ* observation of cell phenotypes, and the released cells are used for genetic analysis, drug screening, gene editing, and biobanks construction.

The establishment of CDX models is time-consuming and inapplicable to large-scale drug screening. CDO is the alternative that allows quick molecular analysis and high-throughput drug screening. An organoid is a special 3D culture model harboring a semisolid extracellular matrix supplemented with growth factors for tissues 71, 72. Organoids have been successfully developed from primary tumors and metastatic lesions from various organs and their practicality has been demonstrated 73, 74. They can be genetically modified using retroviruses, inhibitors, and/or the CRISPR/Cas9 system and can be used to build cancer models and identify key oncogenic “driver mutations” 75-77. More significantly, organoids meet the demand for high-throughput drug screening needed to develop tumor therapeutics 78. As CTCs are rare in the blood, the first successful establishment of CDO models by Gao *et al.* was a significant advance 79. They established seven organoid lines from prostate cancer biopsy specimens and CTCs and reported frequent dysfunction of RB and TP53 pathways in castration-resistant prostate cancer (CRPC) organoid lines, suggesting that drugs targeting these pathways could be promising for therapy. In lung cancer, Zhang *et al.* 80 developed a novel *in situ* capture method for *ex vivo* CTC expansion in a 3D co-culture model simulating tumor microenvironment. They successfully expanded CTCs from 14 out of 19 early-stage lung cancer patients and revealed several mutations, including *TP53* in both cultured CTCs and primary lung cancer. This strategy sets the stage to further characterize CTC biology and metastatic factors in patients with early-stage cancers. Although CDOs have not been established from HCC patients yet, we propose a strategy for analyzing HCC CDOs (**Figure 3**).

## Clinical application in HCC

### Early detection and neoplasm staging

There is considerable evidence for a crucial role of CTCs as initiators of metastasis, suggesting CTCs as a biomarker for the early detection of HCC. In previous CTC studies, early detection of HCC was mainly based on the assessment of CTC numbers, showing a significant positive correlation between the CTC number and the standard Barcelona Clinic Liver Cancer (BCLC) stage as well as the serum AFP level 81. Qi *et al.* used an advanced CanPatrol CTC-enrichment technique and *in situ* hybridization to sort and classify CTCs from blood samples and found 90.18% of HCC patients to be CTC positive, even at the early stage of HCC 31. CTCs were also detected in 2 of 12 patients with hepatitis B virus (HBV) infection, and both patients developed small HCC tumors within 5 months. Another study by Wang* et al.* implicated CTCs in tumor staging 15. The presence of CTCs correlated highly with TNM staging from 66% in stage I to 100% in stage IV with CTC number being 1±1 in stage I to 4±2 in stage IV. Therefore, CTC testing can serve as a supplement to classical TNM staging for improving diagnostic efficiency. However, other studies point out that CTC testing is not an ideal independent diagnostic tool for HCC 82, 83 and simultaneous detection of CTCs and AFP might improve the detection accuracy of HCC patients 83.

### Prognostic evaluation and recurrence monitoring

In patients diagnosed with HCC, CTCs not only contribute to neoplasm staging but are also useful for prognosis 81, 84. Ye *et al.* 85 studied the relationship between the CTC count and the clinical outcome of HCC patients after radical resection and found that both disease-free survival and overall survival were significantly better in patients with lower CTC counts (≤2/5 mL) compared with those with higher counts (>2/5 mL), implicating a high CTC count in the poor prognosis of HCC patients. Yu *et al.* also reported the association of increased postoperative CTC numbers with worse prognosis of HCC patients 86. In a study by Ou *et al.*, CTCs were classified using EMT markers and the presence of mesenchymal CTCs, together with mixed phenotypic and epithelial CTCs predicted the shortest relapse-free survival 87. These observations indicated that the CTC phenotypes might serve as a prognostic indicator for HCC patients, and some specific CTC phenotypes may be more significant for the prognosis than others.

Since CTC numbers may change after anti-tumor treatment, they could be used for predicting or evaluating therapeutic efficacy before or after treatments. Ye *et al.* used CanPatrol™ system to count CTCs 1 day prior to and 30 days after surgical excision of HCC 88. The study showed that postoperative CTC counts (> 2 and > 5) and pre/postoperative change in CTC counts were significantly associated with PFS and were a better predictor of performance than absolute counts. PCR and FACS were used by Zhou *et al.* to detect the preoperative levels of EpCAM^mRNA+^ CTCs and CD4+CD25+Foxp3+ Treg cells in 49 HCC patients. The data showed that elevated CTC/Treg levels implied a higher risk of postoperative recurrence 89.

### Companion diagnostic

Mutation profiles and drug-resistant molecular expression profiles have been widely identified in many solid tumors. In this context, CTC analysis allows the determination of therapeutic targets and resistance mechanisms to cancer therapies at the DNA, RNA, and protein levels and has great potential to identify the patient population most likely to respond to specific treatments. Li *et al.* presented a novel system to provide quantitative information of sorafenib-related targets through simultaneously detecting phosphorylated ERK (pERK) and Akt (pAkt) in HCC CTCs 90. This study demonstrated that CTCs could substitute tumor tissues in the characterization of pERK/pAkt. pERK+/pAkt- CTCs, the most sorafenib-sensitive cells that could serve as an independent PFS indicator in sorafenib-treated HCC patients.

**Figure 4:**
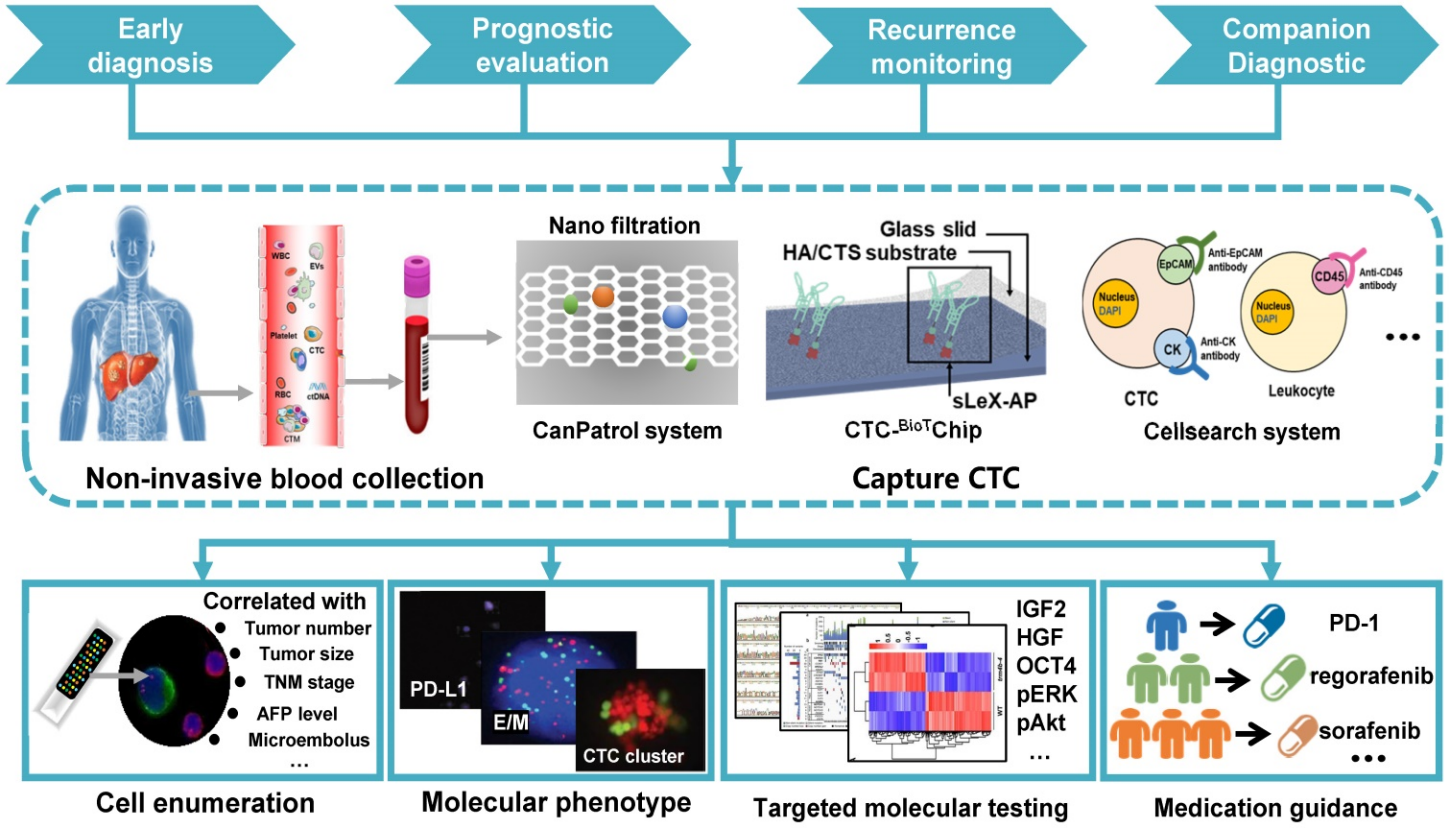
Clinical application of CTCs in HCC: Peripheral blood is obtained from HCC patients using a non-invasive approach, followed by CTC capture on various platforms. Early detection, prognostic evaluation, recurrence monitoring, and companion diagnostic of HCC are achieved by enumerating CTCs, analyzing their molecular phenotypes, and testing targeted genes.

Immune checkpoint inhibitors have launched a new era in immunotherapy, with exceptional long-term remissions in some patients across diverse tumor entities. However, only a few patients respond to this modality, with many experiencing severe side effects 91. Therefore, it is necessary to monitor the expression of molecular targets (such as PD-L1) of immune checkpoint inhibitor therapy. It was first reported by Winograd *et al.* that evaluation of PD-L1+ CTCs discriminated between HCC patients with early-stage and advanced/metastatic disease 92. Of 6 patients receiving anti-PD1 therapy, 3 demonstrated a good response had PD-L1+ CTCs. In contrast, only 1 of 3 non-responders had PD-L1+ CTCs, suggesting that PD-L1+ CTCs might be predictive of immunotherapy response. A more comprehensive clinical application of CTCs in HCC is summarized in **Table 1 and Figure 4.**

## Future perspectives

CTCs offer valuable diagnostic and therapeutic information on HCC, although some challenges still exist in their identification, quantification, and/or characterization. Currently, new CTC capture technologies are emerging, while the CellSearch system is still the only FDA-approved CTC detection and quantification method. So, we urgently need novel and certified tools for quick isolation and characterization of CTCs. There are various sources of CTCs including primary tumors and metastatic lesions. Identifying of the sources of captured CTCs is beneficial for a more in-depth analysis. For a comprehensive understanding of CTC biology, multiple omic disciplines should be combined for single-cell analysis. With the development of CDO approaches, we can co-culture CTCs with immune cells to simulate the tumor microenvironment to monitor tumor progression and associated molecular events. Furthermore, as part of liquid biopsy, CTC testing should be combined with other liquid biopsies such as analysis of ctDNA and exosomes to promote the efficiency of clinical CTC tests. Ongoing and future research on CTC capture technology, molecular profiling, sing-cell analysis, and preclinical models are expected to considerably improve CTC testing for early diagnosis, efficacious treatment, and effective prognostic management of HCC. The overview of CTC research is showed in the **Supplementary Figure.**

## Supplementary Material

Supplementary figures and tables.

## Figures and Tables

**Table 1:** Summary of clinical application of CTCs

Study	Parents	Method	CTC Marker	Main finding	
**Early diagnosis and neoplasm staging**					
Wang S et al 15. 2016	42 HCC	CTC-^BioT^Chip	EpCAM	The platform identified CTCs (2±2 per 2 mL) in 59.5% HCC patients; CTCs were significantly correlated with tumor size, portal vein tumor thrombus, and the TNM (tumor-node-metastasis) stage.	
Qi LN et al 31. 2018	112 HCC treated with R0 resection	CanPatrol^TM^ RNA-ISH	EpCAM, E-cadherin, CK8/18/19, vimentin, Twist	90.18% patients with HCC were CTC positive, even with early-stage disease; CTCs were also detected in 2 of 12 patients with hepatitis B virus (HBV), both of whom had small HCC tumors detected within 5 months.	
**Prognostic evaluation and recurrence monitoring**					
Sun YF et al 93. 2013	123 HCC	CellSearch	EpCAM	CTCs were present in 66.67% of patients; A preoperative CTC 7.5 of ≥2 was an independent prognostic factor for tumor recurrence.	
Yu JJ et al 86. 2018	139 HCC; 23BHT	CellSearch	EpCAM	Patients with increased postoperative CTC counts (from preoperative CTC < 2 to postoperative CTC ≥ 2) had significantly shorter DFS and OS.	
Chen J et al 81. 2017	195 HCC	CanPatrol^TM^	CK, EpCAM, Twist, Cadherin, Snail, Vimentin, AKT2	CTCs were detected in 95% HCC patients. The AUC of the ROC curve was 0.861 in discriminating metastatic and non-metastatic patients. The proportion of hybrid and mesenchymal CTCs was associated with age, BCLC stages, metastasis and AFP levels and recurrence.	
Yin LC et al 84. 2018	80 HCC; 10HV	CanPatrol^TM^	Twist	Twist+ CTCs were detected in 67.5% HCC patients. The positive ratios of Twist+ CTCs correlated with portal vein tumor thrombi, TNM staging, AFP, cirrhosis, tumor number, tumor size, microvascular invasion, as well as postop recurrence and the mortality.	
Ye XP et al 88. 2018	42 HCC	CanPatrol^TM^	TP53	Postoperative CTC counts (> 2 and > 5) and changes in CTC counts may be independent prognostic indicators for PFS in patients with HBV-related HCC	
Shi J et al 94. 2016	47 HCC undergoing cryoablation	MACS, RT-qPCR, MACS, RT-qPCR	MAGE-3, Survivin, CEA	Average CTCs decreased significantly following cryosurgery.	
Zhou Y et al 89. 2016	49 HCC undergoing curative resection; 50 HV	RosetteSep, qRT-PCR	EpCAM, CD4+ CD25+, Foxp3	Patients with high CTC/Treg levels showed a significantly higher risk of developing postoperative HCC and higher 1-year recurrence rates.	
Wang Z et al 95. 2018	62 HCC undergoing radical resection	CanPatrol^TM^	CK, EpCAM, Vimentin, Twist	The total number of CTCs, mesenchymal CTCs, and mixed CTCs in the recurrence group was significantly higher than in the non-recurrence group; Mesenchymal CTCs associated with shortened postoperative DFS.	
Shen J et al 96. 2018	89 HCC undergoing TACE	Cell Search	EpCAM	CTC count is an independent predictor of OS and progression-free survival in patients treated with chemoembolization; A higher number of CTCs associated with mortality and progression.	
von Felden J et al 97. 2017	58 HCC undergoing resection	CellSearch	EpCAM	CTC-positive patients had a significantly higher risk of recurrence with a HR of 2.3, and a shorter RFS.	
Qi LN et al 98. 2018	112 HCC treated with R0 resection; 12 HBV; 20 HV	CanPatrol^TM^	EpCAM, CK, Vimentin, Twist	CTC count ≥16 and mesenchymal-CTC (M-CTC) percentage ≥2% prior to resection was significantly associated with early recurrence, multi-intrahepatic recurrence, and lung metastasis.	
Ou H et al 87. 2018	165 HCC undergoing radical resection	CanPatrol^TM^	EpCAM, CK, Vimentin, Twist	Mesenchymal CTCs were significantly correlated with high AFP levels, multiple tumors, advanced TNM and BCLC stage, presence of embolus or microembolus, and earlier recurrence.	
Sun YF et al 32. 2018	73 HCC undergoing curative resection	CellSearch, qRT-PCR	EpCAM, E-cadherin, N-cadherin, Vimentin, Snail, Slug	CTC and circulating tumor microemboli burden in hepatic veins and peripheral circulation prognosticated postoperative lung metastasis and intrahepatic recurrence, respectively.	
Wang L et al 4. 2018	14 HCC; 16 CCA; 4 GBC undergoing resection	SE-iFISH	aneuploid chromosome 8	Postsurgical quantity of small triploid CTCs (≥5 cells/6 ml blood), multiploid (≥pentasomy 8) CTSCs or CTM (either one ≥ 1) significantly correlated to HCC patients' poor prognosis,	
**Companion Diagnostics**					
Li J et al 90. 2016	109 HCC on sorafenib	Ficoll-Paque	pERK	CTCs could be used in place of tumor tissue for the characterization of pERK/pAkt expression; pERK^+^/pAkt^-^ CTCs were most sensitive to sorafenib and an independent predictive factor of PFS in HCC patients treated with sorafenib.	
Winograd P et al 92. 2018	73 HCC: 8 HV;11 BLD	CTC-iChip	PD-L1	PD-L1 CTC phenotype may help guide selection of patients likely to benefit from immune checkpoint inhibitors.	

BHT, benign hepatic tumor; BLD, benign liver disease; CCA, cholangiocarcinoma; CTC, circulating tumor cell; CTM, circulating tumor microemboli; DFS, disease-free survival; EMT, epithelial to mesenchymal transition; GBC, gallbladder cancer; HBV, hepatitis B virus; HCC, hepatocellular carcinoma; HV, healthy volunteers; IVC, inferior vena cava; LC, liver cirrhosis; OS, overall survival; PFS, progression-free survival; RFS, recurrence-free survival; HR, hazard ratio.

## Abbreviations

AFP: alpha-fetoprotein
ASGPR: asialoglycoprotein receptor
CD44: cluster of differentiation 44
CD45: cluster of differentiation 45
CD47: cluster of differentiation 47
CD90: cluster of differentiation 90
CD133: cluster of differentiation 133
CTC: circulating tumor cell
CTM: circulating tumor microemboli
CDO: CTC-derived organoid
CDX: CTC-derived xenograft
CK19: cytokeratin 19
CTSC: circulating tumor stem cell
CSC: cancer stem cell
CXCR4: chemokine (C-X-C motif) receptor 4
EpCAM: epithelial cell adhesion molecule
EMT: epithelial-mesenchymal transition
GPC3: glypican-3
HBV: hepatitis B virus
HGF: hepatocyte growth factor
HCC: hepatocellular carcinoma
IGF-2: insulin like growth factor 2
Oct4: octamer-binding transcription factor 4
PD‐L1: programmed cell death ligand 1
Sox2: SRY-related high-mobility-group (HMG)-box protein-2
TNM: tumor-node-metastasis
TNBC: triple- negative breast cancer
