# Supplementary material for: Circulating tumor cells in hepatocellular carcinoma: single-cell based analysis, preclinical models, and clinical applications
Source: Theranostics. 2020 Oct 26;10(26):12060–71. doi: 10.7150/thno.48918 (PMC7667686; doi:10.7150/thno.48918)
Supplement: Supplementary file 1 — Supplementary figures and tables. [file thnov10p12060s1.pdf]

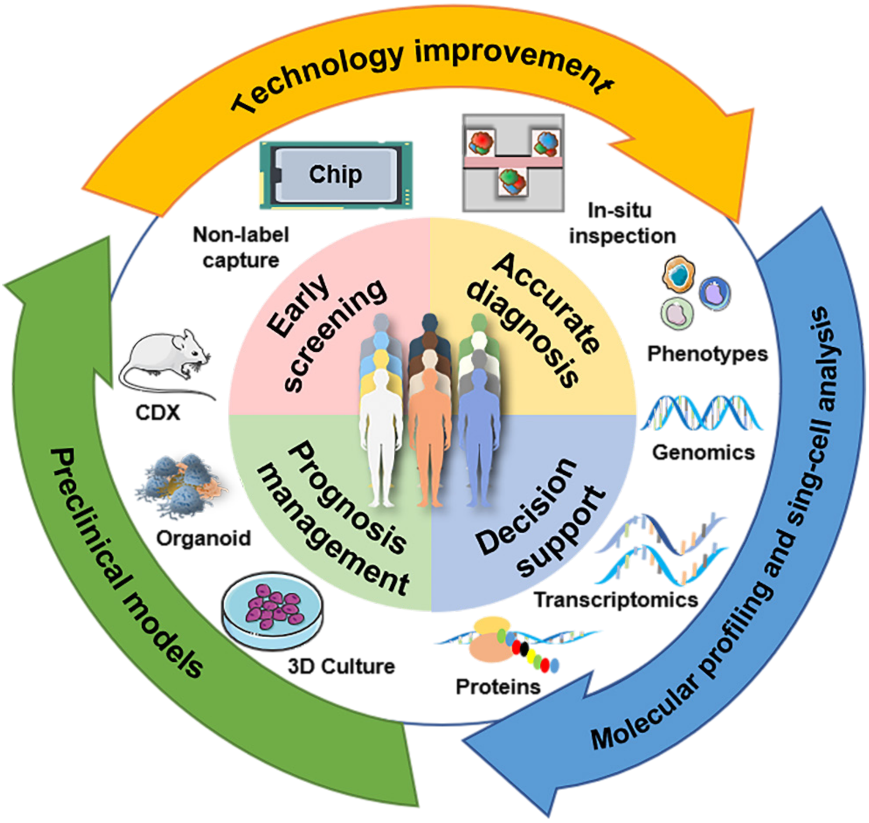

Supplementary figure S1.

Overview of CTC research: The development of CTC capture techniques, molecular profiling methods, single-cell analysis, and CTC-derived clinical models promote the clinical application of CTCs in HCC. There is an urgent need for non-label capture and in-situ inspection methods to facilitate subsequent technology improvement analyses. For molecular profiling and single-cell analysis, the omic technologies should be combined for an in-depth understanding of CTC biology. As for preclinical models, the establishment of CDXs and CDOs in HCC still requires our continued efforts for further improvement.
